# Preparation of Surface-Reinforced Superabsorbent Polymer Hydrogel Microspheres via Incorporation of In Situ Synthesized Silver Nanoparticles

**DOI:** 10.3390/polym13060902

**Published:** 2021-03-15

**Authors:** Semin Kim, Minsu Kim, Won-Gun Koh

**Affiliations:** Department of Chemical and Biomolecular Engineering, Yonsei University, Seoul 03722, Korea; ksmw9292@yonsei.ac.kr (S.K.); coreakms@yonsei.ac.kr (M.K.)

**Keywords:** superabsorbent polymer, surface-reinforced hydrogel particle, silver nanoparticle, gel blocking, micro-CT analysis

## Abstract

Superabsorbent polymer (SAP) particles are primarily applied for absorbing and storing liquids. Here, poly (acrylic acid) (PAA)-based SAP microspheres incorporated with silver nanoparticles (AgNPs) are prepared as an effort to maintain microsphere shape during swelling and minimize gel blocking. PAA-based SAP spheres are synthesized via inverse suspension polymerization. AgNPs are formed within SAP spheres through in situ reduction of silver nitrate (AgNO_3_), using polyvinylpyrrolidone as the reducing agent. The formation of AgNPs within SAP was observed via techniques such as scanning electron microscopy, ultraviolet-visible spectroscopy, thermogravimetric analysis, X-ray photoelectron spectroscopy and transmission electron microscopy. Energy dispersive spectroscopy analyses reveal that thin and dense layers of AgNPs are formed on the outer regions of the SAP spheres at higher concentrations of AgNO_3_. The water absorbency capacity decreases on increasing the amount of incorporated silver nanoparticles; however, it is comparable with that of commercially available surface-crosslinked SAP particles. Finally, micro-computerized tomography (micro-CT) study revealed that AgNP-incorporated SAP spheres maintained their shapes during swelling and exhibit higher void fractions in the packed gel bed, minimizing gel blocking and improving fluid permeability.

## 1. Introduction

Superabsorbent polymers (SAPs) are lightly crosslinked hydrogels capable of absorbing large amounts of water [1,2]. Although SAPs have been extensively investigated and widely applied in various applications such as disposable hygiene [3,4,5], horticultural water retention agent [6,7], drug-delivery systems [8,9] and water-blocking tapes [10,11], a majority of their industrial applications are focused on disposable diapers. Conventionally, partly neutralized, lightly crosslinked poly (acrylic acid) (PAA)-based hydrogel microparticles are used for diaper materials [12,13,14]; they are packed in gel beds to absorb urine. However, owing to their loosely crosslinked nature, original PAA-based SAP microparticles are mechanically weak and undergo rapid swelling [15]. As such, they are unable to absorb liquid effectively, especially when pressure is applied; for example, when a baby sits on a diaper. Furthermore, excessively swollen SAP particles on the top region of the gel bed prevent liquid from entering the interior of the gel bed, which is termed as the gel blocking phenomenon [2,16,17]. These shortcomings as mentioned earlier, resulting from the lightly crosslinked SAP particles, cause diaper leakage and limit the absorption capacity of the gel bed. Thus, efforts have been devoted toward fabricating SAPs that improve water absorption even under pressure and also minimize the gel blocking phenomenon; these efforts have led to the development of surface-crosslinked SAP microparticles [18,19,20,21,22]. Post surface crosslinking of loosely crosslinked PAA-based SAP microparticles creates core-shell structured microparticles consisting of a core with lower crosslinking density and a shell with high crosslinking density [23]. Surface crosslinking enables SAP particles to maintain their shapes during swelling and minimizes gel blocking by generating a less densely packed bed with a void fraction. Therefore, fluid can flow into the bottom region and be absorbed by the SAP particles underneath, enabling a more efficient use of the gel bed. Conventionally, PAA-based SAPs have been thermally or photochemically surface-crosslinked through the formation of an ester or amide linkages [16,24,25,26]. Furthermore, Moini et al. [27] used click chemistry for surface modification of SAP. However, to the best of our knowledge, none of the previous studies have investigated the use of metal nanoparticles to improve the stability and permeability of SAP particles. Although few studies reported the incorporation silver nanoparticles into SAP, they were mainly for antibacterial effects [28,29].

In this study, silver nanoparticles (AgNPs) that could played similar role with conventional surface-crosslinking layer were incorporated into PAA-based SAP spheres. Various analyses were performed to confirm the formation and distribution of the AgNPs within SAP spheres. For the first time, the bulk swelling behavior and void fraction of a gel bed packed with SAP spheres were investigated via micro-computerized tomography (micro-CT). We also evaluated the antibacterial properties of SAP spheres originating from the incorporated AgNPs.

## 2. Materials and Methods

### 2.1. Materials

Acrylic acid (AA), ammonium persulfate (APS), tri (ethylene glycol) diacrylate (TEGDA), cyclohexane, silver nitrate (AgNO_3_), sodium citrate and gold (III) chloride solution (HAuCl_4_) were purchased from Sigma-Aldrich (Milwaukee, WI, USA). Sodium hydroxide (NaOH) was purchased from Ducksan Co., Ltd. (Ansan, Korea). Ethyl cellulose and polyvinylpyrrolidone (PVP) were purchased from JUNSEI Chemicals (Tokyo, Japan) and commercial SAP was purchased from LG Household & Health Care Ltd. (Seoul, Korea). Moreover, *Proteus mirabilis* (ATCC 29906) was purchased from Koram Biotech Co. (Seoul, Korea) and the halo test was commissioned by Korea Standard Test Researcher (KSTR).

### 2.2. Preparation of AgNP-Incorporated SAP Spheres

Spherical SAPs were prepared via inverse suspension polymerization, as described in our previous study [19]. For the dispersed phase, 360 μL of TEGDA and 0.05 g of APS were added to 37 mL of partially neutralized (70%) AA solution to serve as the crosslinking agent and water-soluble initiator, respectively [30]. The partially neutralized AA solution was prepared by adding 25 mL of 5 M NaOH solution to 12 mL of acrylic acid. The previously prepared dispersed phase was then added into a continuous phase, which consisted of 137 mL of cyclohexane and 0.375 g of ethyl cellulose, in a double-wall beaker. While stirring the reagents at 250 rpm, the experiment was conducted for 1 h at 80 °C. Nitrogen gas was continuously purged until all samples were completely prepared to prevent undesired reactions. After the reaction, the prepared SAP spheres were washed overnight in methanol to remove unreacted monomers under vigorous stirring condition and subsequently dried at 25 °C. AgNP-incorporated SAP spheres (AgNP-SAP) were prepared via in situ synthesis of AgNPs in the outer region of the SAP sphere. The SAP spheres (0.2 g) absorbed 30 μL of AgNO_3_ (10 mM, 50 mM and 100 mM) solution and then reacted with the PVP solution (1.667 mM) for 2 h at 150 °C, while being stirred at 300 rpm. After the reaction, AgNP-SAPs were washed overnight in methanol, while being stirred to remove unreacted AgNO_3_ as well as PVP; they were subsequently dried at 25 °C

### 2.3. Water Absorbency Measurement

Water content measurements were conducted via gravimetric analysis of the SAPs and AgNP-SAPs. After the SAPs and AgNP-SAPs were fully swollen and dried, their weights were determined. The SAPs and AgNP-SAPs were first immersed in a 0.9 wt% NaCl solution until fully swollen and then dried overnight in a vacuum oven. To determine the water absorbency, the difference between the dried weight and the swollen weight was divided by the dry weight as shown below:

(1) $$\mathrm{Water}\;\mathrm{absorbency}\;\left[g/g\right]=\;\frac{W_{s}-W_{d}}{W_{d}}$$

where *W_d_* is the dry weight and *W_s_* is the swollen weight.

### 2.4. Characterization Methods

Morphologies of the dried SAP and AgNP-SAPs were observed using a 7610f-Plus field-emission SEM (JEOL Ltd., Tokyo, Japan) equipped with EDS. For the SEM/EDS analysis, the AgNP-SAPs were cut into disk-shaped samples using a pair of razors. Disk-shaped samples were Pt-sputtered for 120 s and the cross-sectional surface images were obtained. TGA (TA Instrument, New Castle, DE, USA) was performed under nitrogen atmosphere to measure the amount of incorporated silver nanoparticles within the SAP spheres. TGA experiments were conducted using the dried AgNP-SAP samples at a heating rate of 10 °C/min in N_2_ condition. The high-resolution analysis was performed via the Lorentz model using Origin 21. UV-Vis absorption spectra of the SAPs and AgNP-SAPs were recorded on a JASCO corporation V-650 model UV-Vis spectrophotometer (Yongin, Korea) with a scan range of 300–600 nm. The sizes of the AgNPs within the AgNP-SAPs were measured using TEM analysis; AgNPs from the AgNP-SAP samples were placed on a copper grid, which was then inserted into a TEM-F200 model (JEOL Ltd., Tokyo, Japan) for the TEM analysis. In this analysis of UV-vis and TEM, as in our previous study [31], finely ground SAP and AgNP-SAPs samples were dispersed in 3 mL of DI water and allowed to soak for one day, to take out the AgNPs from the SAP network to the aqueous phase.

### 2.5. Analyses of Bulk Swelling Behavior of Packed SAP Spheres

Bulk swelling properties of the packed SAP spheres were monitored using micro-CT (Bruker-CT, Kartuizersweg 3B 2550 Kontich, Belgium). The cylindrical Styrofoam mold was filled with dried SAPs or AgNP-SAPs, which were then fully swollen with 0.9 wt% NaCl solution. The cylindrical molds filled with dried and swollen SAPs were photographed using a micro-CT and 3D images of the SAP-packed molds were obtained. Micro-CT was also used to measure both the cylindrical mold and SAP system volumes, which allowed us to calculate the void volume fraction of packed SAP systems using the following equation:

(2) $$\mathrm{Void}\;\mathrm{volume}\;\mathrm{fraction}\;\mathrm{of}\;\mathrm{SAP}\;\left[\%\right]=\;\frac{V_{total}-V_{SAP}}{V_{total}}\times 100$$

where *V_total_* is the volume of SAPs, including the inter-SAP space and *V_SAP_* is the volume of SAPs. To evaluate gel-blocking inhibition, 0.9 wt% gold nanoparticle (AuNP) solution was added to the swollen SAPs and AgNP-SAPs and then packed in a cylindrical mold. AuNPs were prepared as described in a previous study [32]. The resultant AuNPs exhibited a negative charge owing to the citrate group; they were electrostatically coated with 1.5 wt% bovine serum albumin to improve their stability in the aqueous solution.

### 2.6. Antibacterial Test

A halo test was conducted by a Korea Standard Test Researcher (KSTR, Gwacheon, Korea) in order to investigate the antibacterial effects of the incorporated AgNPs. For the CFU method, samples were exposed to bacteria in solid media (SAP and AgNP-SAP). *Proteus mirabilis* (ATCC 29906) was incubated in the control SAP and AgNP-SAP for 12 h. Thereafter, the number of initial bacteria and the number of bacteria after incubation were compared.

## 3. Results and Discussion

### 3.1. Characterization of SAPs and AgNP-SAPs

**Figure 1:**
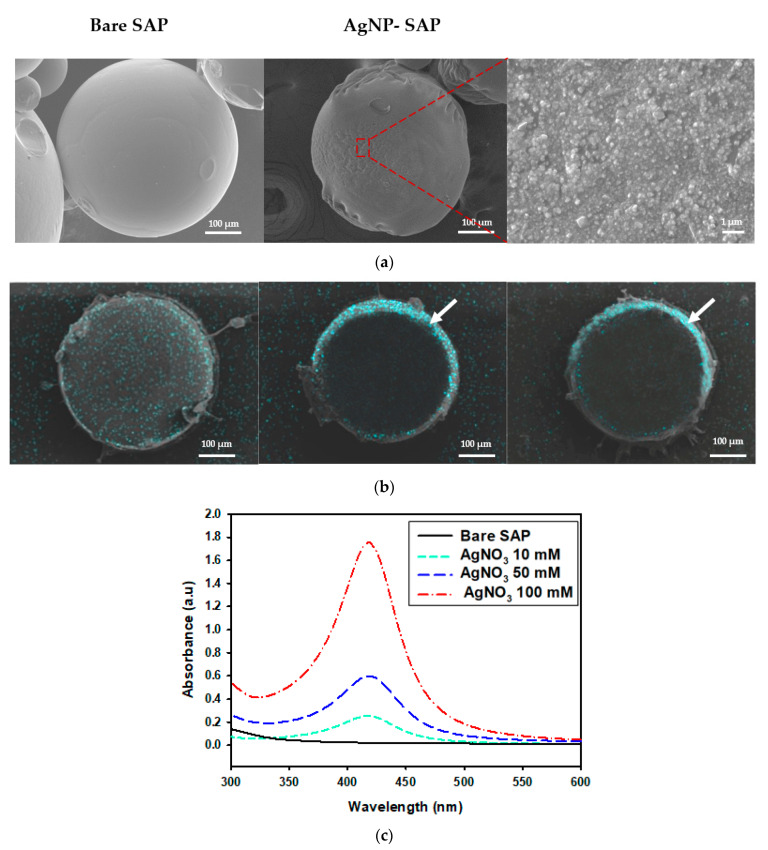
Characterization of different SAP spheres. (**a**) SEM images of the surface of bare SAP and AgNP-SAP prepared from 50 mM AgNO_3_. (**b**) EDS mapping images for silver element obtained from cross-section of AgNP-SAP (sky blue that arrows indicate is the layer of AgNPs). (**c**) UV-Vis spectra of AgNP-SAP prepared from different concentration of AgNO_3_.

First, SAP spheres of 400 μm-diameter were synthesized via inverse suspension polymerization, where partially (70%) neutralized acrylic acid and tri-ethylene glycol diacrylate (TEGDA) were used as the monomers and the crosslinkers, respectively. AgNPs were incorporated into the SAPs via AgNO_3_ absorption and subsequent Ag^+^ reduction using polyvinylpyrrolidone (PVP) as the reducing agent. Scanning electron microscope (SEM) images indicate that bare SAPs feature a smooth surface, whereas AgNP-SAPs exhibit rough surfaces owing to the presence of AgNPs (Figure 1a). The distribution of AgNPs within the SAP was analyzed via an SEM equipped with energy dispersive spectroscopy (SEM/EDS), as shown in Figure 1b. At a low concentration of AgNO_3_ (10 mM), few AgNPs were distributed evenly over the entire sample. As the AgNO_3_ concentration increased, most of the AgNPs moved to the outer regions of the SAP spheres, where a higher concentration of AgNO_3_ resulted in the production of additional AgNPs in the form of thinner and denser layers. First, AgNO_3_ diffused into SAP and Ag^+^ distributed evenly inside SAP spheres via electrostatic interaction with COO^−^ in PAA, which were then reduced by PVP to form AgNPs. If a small amount of AgNO_3_ was present in the SAP, PVP easily diffused into SAP spheres, producing AgNPs which were evenly distributed inside the SAP spheres. However, due to the low concentration of AgNO_3_, density of AgNPs within SAP was relatively low. When a high concentration of AgNO_3_ filled the free volume of SAP, the diffusion of PVP was limited and PVP could not penetrate deep into the SAP spheres. Therefore, most of the reduction reaction occurred in the outer region of SAP spheres. Since the concentration of AgNO_3_ was high, a larger amount of AgNPs were produced to form a dense layer in outer region. According to EDS analysis, atomic % of silver was 0.5, 1.85 and 2.37%, respectively, when the concentration of AgNO_3_ was 10, 50 and 100 mM, demonstrating that more AgNPs were incorporated into SAP with the increase of AgNO_3_ concentration.

The presence of silver nanoparticles in AgNP-SAPs was verified via UV-Vis spectrophotometry. According to the UV-Vis spectra shown in Figure 1c, the AgNP-SAPs feature a distinct absorbance peak near 423 nm resulting from the surface plasmon resonance of AgNPs [33], the intensity of which increased with the concentration of AgNO_3_. As more amount of AgNO_3_ was used, silver salt loading within the SAP sphere increased, resulting in more reduction reaction to produce additional AgNP within the SAP sphere. The formation of more AgNPs increased the absorbance indicated in the UV-Vis spectra. These data confirmed that AgNPs were successfully formed within the SAP spheres, where the location and the amount of AgNPs were dependent on the amount of AgNO_3_.

**Figure 2:**
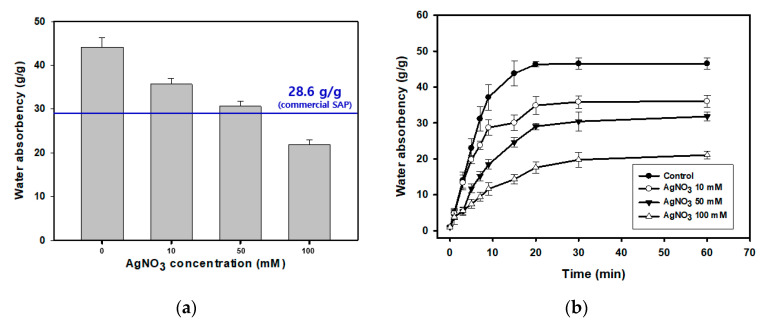
Water absorbency behavior of AgNP-SAP. (**a**) Water absorbency at equilibrium. (**b**) Swelling rate of AgNP-SAP prepared from different concentration of AgNO_3_ (0.9 wt% NaCl solution was used).

The incorporation of AgNPs significantly changed the swelling property of SAPs in terms of water absorbency and swelling rate. Experiments conducted using the centrifuge retention capacity process revealed that water absorbency decreased proportionally with the increasing concentration of AgNO_3_ (Figure 2a). The incorporated AgNPs occupied the free volume in the SAPs, effectively decreasing the available SAP volume for absorbing water; they also significantly increased the dry weight of the SAP. The water absorbency of commercial SAPs is 28.6 ± 1.6 g/g, as indicated by the blue line in Figure 2a; this is similar to that of the AgNP-SAP prepared using 50 mM AgNO_3_. Thus, the remaining experiments were performed using AgNP-SAP prepared with 50 mM AgNO_3_. The swelling rate became slower and the total water absorbency decreased as the amount of AgNP in SAP sphere increased as shown in Figure 2b.

**Figure 3:**
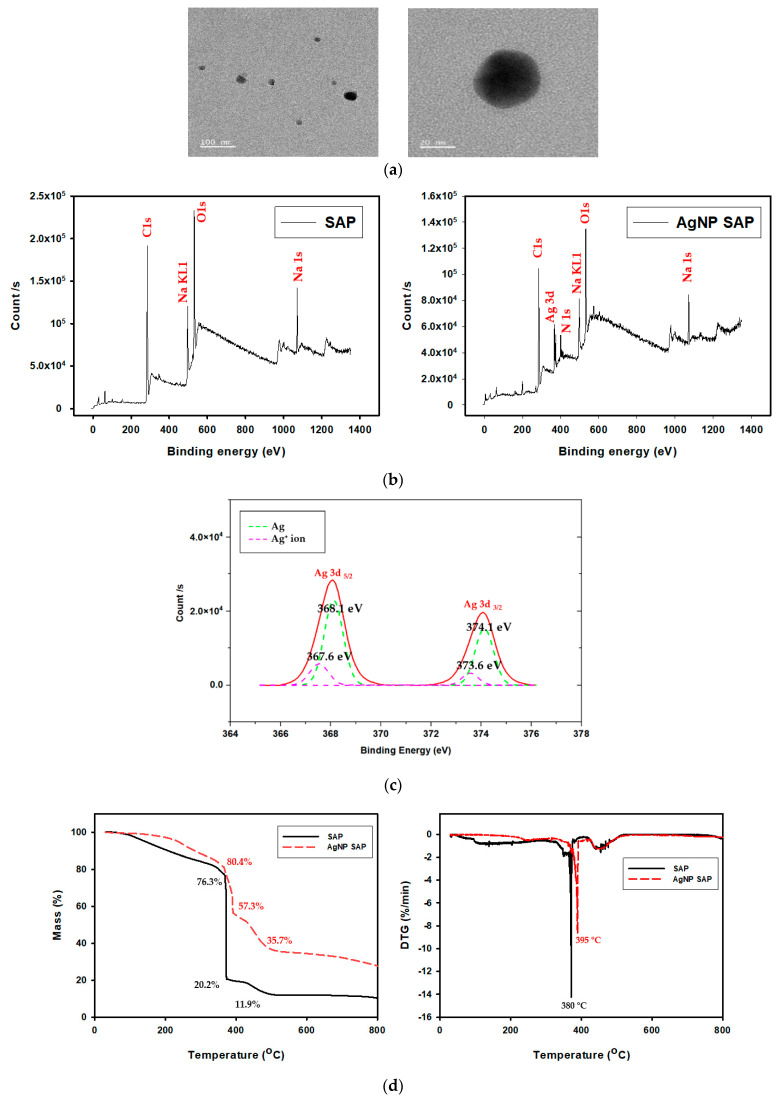
(**a**) TEM image of AgNPs produced in SAPs. (**b**) XPS spectra of bare SAP and AgNP-SAP. (**c**) High resolution XPS Ad3d spectra for the AgNP-SAP. (**d**) TGA and DTGA thermogram of SAP and AgNP-SAP.

The transmission electron microscopy (TEM) image in Figure 3a reveals that the AgNPs formed in the SAP spheres are spherical, with diameters of 20–30 nm. Embedded AgNPs within the SAP network were also observed using X-ray photoelectron spectroscopy (XPS). Figure 3b presents the surface-scanned XPS spectra obtained from the SAP and AgNP-SAPs. Na1s peak in both samples was originated from COO^-^Na^+^ in PAA chain after neutralizing with NaOH, while The N1s peak newly observed from AgNP-SAP was originated from the PVP bound to AgNPs. High resolution XPS scan over Ag3d was shown in Figure 3c to distinguish metallic silver and silver ions. According to the Figure 3c, binding energy of 368.1 eV and 374.1 eV corresponded to metallic silver (Ag), while 367.6 eV and 373.6 eV to silver ions (Ag^+^) [34,35]. Ratio of ionic to metallic silver was 0.26:1. From the dominant peak for metallic silver, it was confirmed that Ag^+^ was successfully reduced to AgNPs by PVP.

Thermogravimetric analysis (TGA) (Figure 3d) conducted under N_2_ condition. Therefore, remained weight in bare SAP sample can be regarded as charred organics leftovers and the difference between bare and AgNP-SAP as silver amount. The first mass loss observed below 380 °C resulted from the evaporation of water. The second loss at around 380 °C was from the decomposition of sodium polyacrylate crosslinks, while the third loss between 400 °C and 480 °C was attributed to decomposition of PAA and PVP [36,37,38]. Since it was known that silver nanoparticles remained at temperatures below 800 °C, remaining mass was due to the silver nanoparticles formed in AgNP-SAP [38]. By referring to the morphology and chemical analysis results, we can confirm that spherical metallic AgNPs were successfully prepared within the SAP through in situ reduction processes.

### 3.2. Analysis of Gel Blocking Phenomenon

The primary application of SAPs in the particle form is for the absorption and storage of liquids in hygienic products such as diapers. For such applications, packed SAP particles are employed and gel blocking is undesirable. Gel blocking is a phenomenon that occurs when excessive swelling of SAP particles in the outer region blocks the passage of fluid into the inner region, thereby reducing the absorption capacity of the SAP. In this work, the swelling properties of the packed SAP spheres were monitored via micro-CT. After Styrofoam mold was packed with swollen SAPs or AgNP-SAP, micro-CT images were obtained, where gray and black region represented SAP and void volume between SAP spheres, respectively.

**Figure 4:**
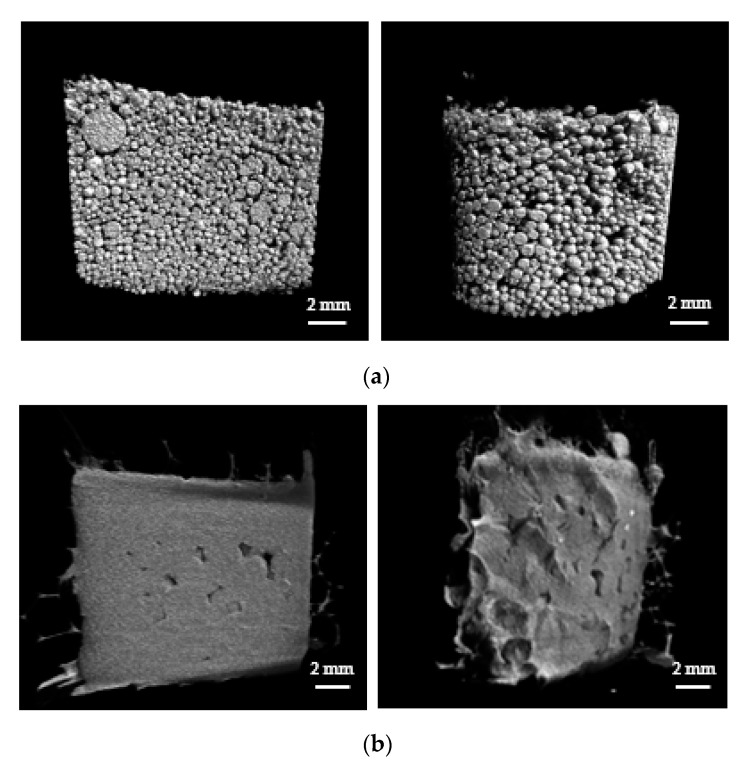

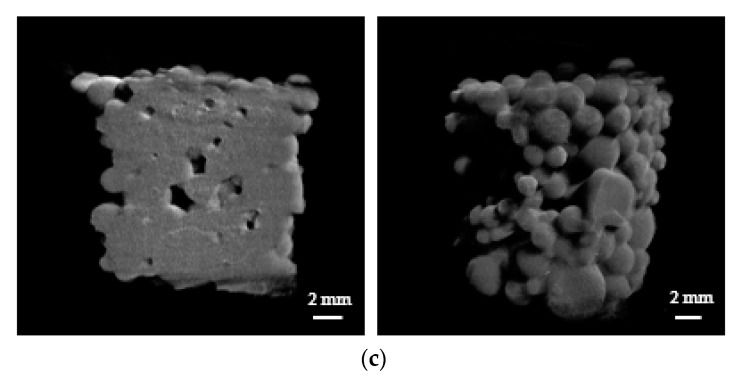
Micro-CT analysis (left: cross-section region, right: outer region) on the swelling properties of the packed SAP spheres. Micro-CT images of (**a**) dried SAPs (**b**) swollen SAPs and (**c**) swollen AgNP-SAPs packed in the mold.

**Figure 5:**
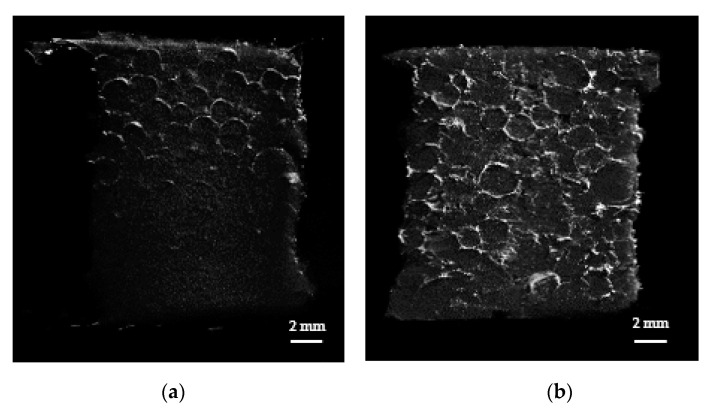
Permeability of gel bed packed with (**a**) SAPS and (**b**) AgNP-SAPs. Severe gel blocking was only observed in SAP-packed mold, while AgNP-SAPs showed good permeability.

Defined volume within the mold was selected and used for the calculation of void volume fraction. Prior to the exposure to water, a lot of SAP spheres filled the mold and the void volume fraction for SAPs was approximately 40% (Figure 4a). The exposure to water caused swelling of both the SAPs and the AgNP-SAPs. Notably, the SAPs underwent excessive swelling such that each particle lost its original spherical shape. Moreover, it appears that different particles were merged to form a single massive hydrogel, which decreased the void volume fraction to 10.92% (Figure 4b). However, the AgNP-SAPs retained their spherical shape despite the water absorption (Figure 4c). The void volume fractions of the SAPs and the AgNP-SAPs changed from 10.09 ± 1.46% to 20.30 ± 3.34%. These results demonstrated that the dense layer of AgNPs in the SAPs acted as a surface in situ shell in the conventional SAP particles, which provided mechanical stability to the SAPs and effectively prevented the excessive swelling of the SAPs. Consequently, the gel-blocking phenomenon in the packed AgNP-SAPs was minimized, as compared to the bare SAPs. To analyze the gel blocking phenomenon via micro-CT, gold nanoparticles (AuNPs) solution (bright portion) was poured to the mold packed with swollen SAP and AgNP-SAP; the images thus obtained are shown in Figure 5a,b.

For the bare SAP, most of the AuNPs solution was restricted to the upper region and could not infiltrate the bottom region, because excessive swelling at the top layer blocked the downward flow of the solution. On the contrary, for the AgNP-SAPs, the AuNPs solution could infiltrate the bottom layer of the AgNP-packed cylindrical mold as well as the void spaces in the upper and lower regions. Thus, the surface incorporated AgNPs acted as prevented the excessive swelling of the SAP, thereby alleviating the gel blocking phenomenon.

### 3.3. Amtibacterial Test of AgNP-SAPs

As AgNPs are well-known for their strong antimicrobial activity, we investigated the antibacterial effect of incorporating AgNPs in SAPs. *Proteus mirabilis* was chosen as the model bacterium as it can cause urinary tract infections. 3.5 × 10^3^ CFU/mL of *proteus mirabilis* was inoculated into a petri dish containing AgNP-SAPs prepared with different AgNO_3_ concentrations (0, 10, 50 and 100 mM). The number of bacteria after 12 h of incubation was 2.4 × 10^7^ CFU/mL for the bare SAPs and less than 1.0 CFU/mL for the AgNP-SAPs, regardless of the AgNO_3_ concentrations (Table 1). Therefore, it was concluded that the AgNP layer in SAPs offers antibacterial benefits, in addition to acting as a surface shell.

## 4. Conclusions

In this work, we report on the preparation of acrylic acid-based SAP spheres that incorporate AgNPs. SAP spheres were prepared via the synthesis of poly (acrylic acid) hydrogels through inverse suspension polymerization. The SAP spheres were transformed into AgNP-SAPs through the addition of AgNO_3_ and PVP (as a reducing agent), which resulted in the in situ formation of AgNPs within SAP microspheres. When a higher concentration of AgNO_3_ was used, thin and dense layers of AgNPs were obtained at the outer regions of the SAP spheres, generating a two-layered structure comprising a bare SAP core and an AgNP-incorporated shell. The AgNP layer improved the mechanical stability of the SAPs and helped maintain the shapes of the SAPs during swelling; this enabled additional void volume fractions and eventually minimized the gel blocking phenomenon in a packed SAP particle system. We demonstrated that the incorporation of AgNPs into SAP spheres improves their physical properties and also offers antibacterial benefits.

## Figures and Tables

**Table 1:** Antibacterial test using *Proteus mirabilis* (ATCC29906).

Proteus mirabilis ATCC 29906	Control	AgNO_3_ 10 mM	AgNO_3_ 50 mM	AgNO_3_ 100 mM
Number of bacteria after 12 h (CFU/mL)	2.4 × 10^7^	<1	<1	<1
Concentration of inoculum	3.5 × 10^3^ CFU/mL			

## Data Availability

The data presented in this study are available on request from the corresponding author. At the time the project was carried out, there was no obligation to make the data publicly available.
